# Modification of Huntington’s disease by short tandem repeats

**DOI:** 10.1093/braincomms/fcae016

**Published:** 2024-01-23

**Authors:** Eun Pyo Hong, Eliana Marisa Ramos, N Ahmad Aziz, Thomas H Massey, Branduff McAllister, Sergey Lobanov, Lesley Jones, Peter Holmans, Seung Kwak, Michael Orth, Marc Ciosi, Vilija Lomeikaite, Darren G Monckton, Jeffrey D Long, Diane Lucente, Vanessa C Wheeler, Tammy Gillis, Marcy E MacDonald, Jorge Sequeiros, James F Gusella, Jong-Min Lee

**Affiliations:** 1 Molecular Neurogenetics Unit, Center for Genomic Medicine, Massachusetts General Hospital, Boston, MA 02114, USA; Department of Neurology, Harvard Medical School, Boston, MA 02115, USA; Medical and Population Genetics Program, The Broad Institute of M.I.T. and Harvard, Cambridge, MA 02142, USA; 1 Molecular Neurogenetics Unit, Center for Genomic Medicine, Massachusetts General Hospital, Boston, MA 02114, USA; Department of Neurology, Harvard Medical School, Boston, MA 02115, USA; Population & Clinical Neuroepidemiology, German Center for Neurodegenerative Diseases, 53127 Bonn, Germany; Department of Neurology, Faculty of Medicine, University of Bonn, Bonn D-53113, Germany; Centre for Neuropsychiatric Genetics and Genomics, Division of Psychological Medicine and Clinical Neurosciences, School of Medicine, Cardiff University, Cardiff CF24 4HQ, UK; Centre for Neuropsychiatric Genetics and Genomics, Division of Psychological Medicine and Clinical Neurosciences, School of Medicine, Cardiff University, Cardiff CF24 4HQ, UK; Centre for Neuropsychiatric Genetics and Genomics, Division of Psychological Medicine and Clinical Neurosciences, School of Medicine, Cardiff University, Cardiff CF24 4HQ, UK; Centre for Neuropsychiatric Genetics and Genomics, Division of Psychological Medicine and Clinical Neurosciences, School of Medicine, Cardiff University, Cardiff CF24 4HQ, UK; Centre for Neuropsychiatric Genetics and Genomics, Division of Psychological Medicine and Clinical Neurosciences, School of Medicine, Cardiff University, Cardiff CF24 4HQ, UK; Molecular System Biology, CHDI Foundation, Princeton, NJ 08540, USA; University Hospital of Old Age Psychiatry and Psychotherapy, Bern University, CH-3000 Bern 60, Switzerland; School of Molecular Biosciences, College of Medical, Veterinary and Life Sciences, University of Glasgow, Glasgow G12 8QQ, UK; School of Molecular Biosciences, College of Medical, Veterinary and Life Sciences, University of Glasgow, Glasgow G12 8QQ, UK; School of Molecular Biosciences, College of Medical, Veterinary and Life Sciences, University of Glasgow, Glasgow G12 8QQ, UK; Department of Psychiatry, Carver College of Medicine and Department of Biostatistics, College of Public Health, University of Iowa, Iowa City, IA 52242, USA; 1 Molecular Neurogenetics Unit, Center for Genomic Medicine, Massachusetts General Hospital, Boston, MA 02114, USA; 1 Molecular Neurogenetics Unit, Center for Genomic Medicine, Massachusetts General Hospital, Boston, MA 02114, USA; Department of Neurology, Harvard Medical School, Boston, MA 02115, USA; 1 Molecular Neurogenetics Unit, Center for Genomic Medicine, Massachusetts General Hospital, Boston, MA 02114, USA; 1 Molecular Neurogenetics Unit, Center for Genomic Medicine, Massachusetts General Hospital, Boston, MA 02114, USA; Department of Neurology, Harvard Medical School, Boston, MA 02115, USA; Medical and Population Genetics Program, The Broad Institute of M.I.T. and Harvard, Cambridge, MA 02142, USA; UnIGENe, IBMC—Institute for Molecular and Cell Biology, i3S—Instituto de Investigação e Inovação em Saúde, Universidade do Porto, Porto 420-135, Portugal; ICBAS School of Medicine and Biomedical Sciences, University of Porto, Porto 420-135, Portugal; 1 Molecular Neurogenetics Unit, Center for Genomic Medicine, Massachusetts General Hospital, Boston, MA 02114, USA; Medical and Population Genetics Program, The Broad Institute of M.I.T. and Harvard, Cambridge, MA 02142, USA; Department of Genetics, Blavatnik Institute, Harvard Medical School, Boston, MA 02115, USA; 1 Molecular Neurogenetics Unit, Center for Genomic Medicine, Massachusetts General Hospital, Boston, MA 02114, USA; Department of Neurology, Harvard Medical School, Boston, MA 02115, USA; Medical and Population Genetics Program, The Broad Institute of M.I.T. and Harvard, Cambridge, MA 02142, USA

**Keywords:** Huntington’s disease, genetic modification, polyglutamine disease, *ATXN3*, short tandem repeat

## Abstract

Expansions of glutamine-coding CAG trinucleotide repeats cause a number of neurodegenerative diseases, including Huntington’s disease and several of spinocerebellar ataxias. In general, age-at-onset of the polyglutamine diseases is inversely correlated with the size of the respective inherited expanded CAG repeat. Expanded CAG repeats are also somatically unstable in certain tissues, and age-at-onset of Huntington’s disease corrected for individual *HTT* CAG repeat length (i.e. residual age-at-onset), is modified by repeat instability-related DNA maintenance/repair genes as demonstrated by recent genome-wide association studies. Modification of one polyglutamine disease (e.g. Huntington’s disease) by the repeat length of another (e.g. *ATXN3,* CAG expansions in which cause spinocerebellar ataxia 3) has also been hypothesized. Consequently, we determined whether age-at-onset in Huntington’s disease is modified by the CAG repeats of other polyglutamine disease genes. We found that the CAG measured repeat sizes of other polyglutamine disease genes that were polymorphic in Huntington’s disease participants but did not influence Huntington’s disease age-at-onset. Additional analysis focusing specifically on *ATXN3* in a larger sample set (*n* = 1388) confirmed the lack of association between Huntington’s disease residual age-at-onset and *ATXN3* CAG repeat length. Additionally, neither our Huntington’s disease onset modifier genome-wide association studies single nucleotide polymorphism data nor imputed short tandem repeat data supported the involvement of other polyglutamine disease genes in modifying Huntington’s disease. By contrast, our genome-wide association studies based on imputed short tandem repeats revealed significant modification signals for other genomic regions. Together, our short tandem repeat genome-wide association studies show that modification of Huntington’s disease is associated with short tandem repeats that do not involve other polyglutamine disease-causing genes, refining the landscape of Huntington’s disease modification and highlighting the importance of rigorous data analysis, especially in genetic studies testing candidate modifiers.

## Teaser

Hong *et al*. determined whether polyglutamine disease-associated CAG repeats modify Huntington's disease (Huntington’s disease). Analysis of typed and imputed tandem repeats did not support significant associations between the sizes of CAG repeat of other polyglutamine disease-causing genes and Huntington’s disease age-at-onset while revealing potential modification signals for tandem repeats at other loci.

## Graphical Abstract

**Graphical Abstract:**
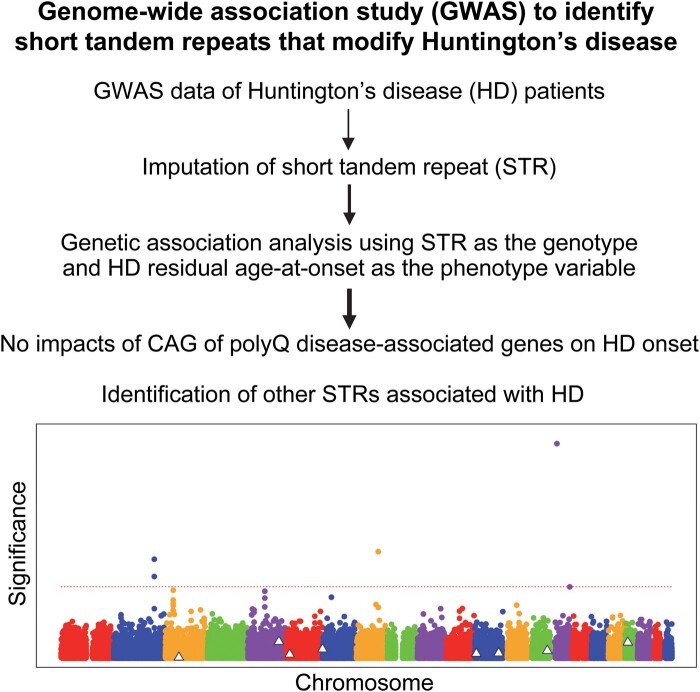

See J. Hannan (https://doi.org/10.1093/braincomms/fcae047) for a scientific commentary on this article.

## Introduction

Expansions of glutamine-encoding cytosine-adenine-guanine (CAG) trinucleotide repeats cause at least nine neurodegenerative diseases, including Huntington’s disease (HD; MIM #143100), several spinocerebellar ataxias (SCAs) and dentatorubral-pallidoluysian atrophy (DRPLA).^1-3^ The polyglutamine expansion diseases exhibit differences in pathogenesis, susceptible brain regions and disease symptoms.^4,5^ However, they share a common feature of inverse correlation between age-at-onset and the length of the causative expanded CAG repeat,^5-12^ indicating that increases in CAG repeat size result in accelerated pathogenesis. However, variance in age-at-onset is not fully explained by the glutamine-encoding CAG repeat length alone. For example, the residual variance in HD age-at-onset (i.e. not due to CAG repeat size) showed heritability,^13^ prompting genome-wide association studies (GWAS) to identify genetic modifiers of HD. Several genetic loci discovered to influence HD age-at-onset harbour DNA repair genes, such as *MLH1, MSH3* and *FAN1*.^14,15^ These genes have been associated with somatic instability of *HTT* CAG repeats in humans and model systems.^16-25^ There is also evidence that these and other DNA repair genes may influence somatic CAG repeat expansions and impact other repeat expansion disorders.^16-19,21,26-39^ The striatum, which is severely affected in HD, shows the highest levels of somatic *HTT* CAG repeat expansion; however, expanded repeats in other polyglutamine diseases can also undergo CAG expansion in this brain region.^40-44^ Interestingly, candidate gene studies have reported modification of HD by normal CAG repeats in *ATXN3* (expansions which are responsible for SCA3),^45^ and conversely modification of SCA3 by the normal *HTT* CAG repeat.^46^ Although *HTT* and *ATXN3* have potential roles in the DNA damage response,^37,47^ neither *HTT* nor *ATXN3* are known to be directly involved in DNA repair. Therefore, this mutual modification has suggested the possibility of a novel mechanism underlying polyglutamine diseases. Consequently, using a variety of genomic data, we set out to determine whether HD is modified by CAG repeat length in other polyglutamine disease genes or other short tandem repeats (STR).

## Materials and methods

### Study subjects

To identify genetic modifiers of HD motor onset, a total of 9058 HD subjects (carrying inherited CAG 40 to 55) of European ancestry were previously analyzed in our GWAS.^15^ Among those HD subjects, we analyzed participants of the COHORT study (https://clinicaltrials.gov/ct2/show/NCT00313495) to test association between HD age-at-onset and experimentally determined (i.e. genotyped) CAG repeat lengths of other polyglutamine disease-causing genes (*n* = 606). For *ATXN3*-focused analysis, we also analyzed participants of the REGISTRY study (https://clinicaltrials.gov/ct2/show/NCT01590589) (*n* = 885). Details of study approval, genotyping, determination of CAG repeat size and calculation of residual age-at-onset are described elsewhere.^15^

### Determination of the CAG repeats in the COHORT and REGISTRY samples

We determined the sizes of CAG repeats of *ATN1* [DRPLA MIM #125370]*, ATXN1* [spinocerebellar ataxia type 1; SCA1 MIM #164400]*, ATXN2* [spinocerebellar ataxia type 2; SCA2 MIM #183090]*, ATXN3* [Machado-Joseph disease aka spinocerebellar ataxia type 3; SCA3 MIM #109150]*, CACNA1A* [spinocerebellar ataxia type 6; SCA6 MIM #183086] and *TBP* [spinocerebellar ataxia type 17; SCA17 MIM #607136] in the participants of the COHORT study (*n* = 606). After quality control, 551, 502, 604, 503, 497 and 483 COHORT samples were analyzed for *ATN1*, *ATXN1*, *ATXN2*, *ATXN3*, *CACNA1A* and *TBP*, respectively. In addition, REGISTRY samples were analyzed to determine the CAG repeat sizes of *ATXN3* (*n* = 885). CAG repeat lengths for each polyglutamine disease-causing genes were determined by polymerase chain reaction (PCR) assays, using fluorescently labelled primers with minor modifications.^48^ PCR products were resolved by an ABI PRISM 3730XL automated DNA Sequencer (Applied Biosystems) and analyzed using GeneMapper version 3.7 software. A set of genomic DNA standard samples were also sequenced for each polyglutamine disease-causing repeat and used as references of CAG allele sizes. Expansions above the non-disease associated repeat range were sequenced after gel separation to further confirm the number of CAGs and the presence of CAA/CAT interruptions.

### Analysis to determine the modification of HD by the CAG repeats of other polyglutamine disease-causing genes

Residual age-at-onset of HD, representing age-at-onset corrected for individual *HTT* CAG repeat length, was based on the rater's estimation of onset age of motor symptoms and the uninterrupted *HTT* CAG repeat size.^12,15^ For example, an HD subject with a positive residual age-at-onset of five means that the individual developed motor onset 5 years later than expected based on his or her uninterrupted CAG repeat size. To determine whether CAG repeat sizes of other polyglutamine disease-causing genes modify HD age-at-onset, we used the same residual age-at-onset phenotype used in our GWAS to identify onset modifiers of HD.^15,49^ Briefly, we modelled residual age-at-onset of HD as a function of the CAG repeat of another polyglutamine disease gene with a set of covariates including four genotype-based principal components, sex, and study group in a linear regression analysis. To analyze typed CAG repeats of each polyglutamine disease gene, three separate linear regression models were constructed to test the longer, the shorter, and the sum of both repeat lengths. To validate previous dichotomous *ATXN3* CAG repeat association analysis,^45^ we also performed a Mann–Whitney *U* test to compare age-at-onset and residual age-at-onset between HD subjects carrying below versus above the median of longer *ATXN3* CAG repeat (i.e. 23 CAG). Specifically, based on the longer of two *ATXN3* CAG repeats in a given individual (i) HD subjects carrying 22 or shorter repeats were assigned as below the median group, and (ii) HD subjects with 24 or higher repeats were assigned as above the median group for the dichotomous analysis.

### SNP association analysis for other polyglutamine disease-causing genes in HD modifier GWAS data

We evaluated the levels of association between residual age-at-onset of HD and other polyglutamine disease-causing genes by checking SNP data available at the GeM Euro 9 K website (cegeme.partners.org/gem.euro.9k).^49^ For each gene, we took a region for the RefSeq select transcript and identified the SNP with the highest significance in our HD subject-based GWA analysis to assess the levels of modification of HD by other polyglutamine disease genes.

### Imputation of STR lengths from GWAS SNP data and association analysis

For the imputation of STR lengths in our GWAS data, we performed quality control analysis of typed genotype data by taking SNPs with call rate >95% and minor allele frequency >1%. Allele frequencies of SNPs and reference alleles in the typed data set were compared to those of 1000 Genomes Project data to confirm data quality using the conform-gt (https://faculty.washington.edu/browning/conform-gt.html). Then, imputation of autosomal STRs was performed by the Beagle programme (https://faculty.washington.edu/browning/beagle/beagle.html; v4.1) using the 1000 Genomes Project reference panel consisting of SNPs and STRs.^50,51^ Imputed STR data were further filtered by taking tandem repeats located by the ‘Tandem Repeats Finder’ algorithm^52^ and annotated as ‘SimpleRepeat’ in the University of California, Santa Cruz (UCSC) genome browser (https://genome.ucsc.edu/index.html). These procedures generated repeat length genotypes of 66 154 tandem repeats for the 9058 HD subjects. We finally selected 58 894 tandem repeats that were polymorphic in our data for the subsequent association analysis. The proportions of repeats of 1, 2, 3, 4, 5 and 6 nucleotide motif were 0.30, 48.89, 9.47, 29.37, 7.84 and 2.38%, accounting for 98.6% of all analyzed tandem repeats. For association analysis, the sum of two repeat sizes was used as the independent variable (which was similar to the additive model of single SNP association analysis) with the same covariates that were used in our SNP GWAS to explain residual age-at-onset.^15^

## Results

### No significant association between HD and CAG repeat lengths of other polyglutamine disease-causing genes

To determine whether age-at-onset in HD is modified by the CAG repeats of other polyglutamine disease-causing genes, we determined directly the length of CAG repeats in *ATN1* (DRPLA), *ATXN1* (SCA1), *ATXN2* (SCA2)*, ATXN3* (SCA3), *CACNA1A* (SCA6) and *TBP* (SCA17) in the HD individuals who participated in both the COHORT study (https://clinicaltrials.gov/ct2/show/NCT00313495) and our recent HD modifier GWA analysis.^15^ The CAG repeat sizes of the polyglutamine disease-causing genes showed distinct distribution patterns. For example, *CACNA1A* and *TBP* showed the smallest and the largest median repeat sizes, while *ATXN2* and *ATXN3,* respectively showed different ranges of repeat lengths despite similar median repeat sizes (Supplementary Fig. 1). Next, we performed statistical analyses of HD subjects with European ancestry to determine whether (i) the longer repeat, (ii) the shorter repeat, or (iii) the sum of the two repeat alleles of other polyglutamine disease-causing genes were associated with residual age-at-onset of HD. As the primary phenotype of the analysis, we used residual age-at-onset of HD motor symptoms representing age-at-onset that was corrected for individual pathogenic *HTT* CAG repeat size. In linear regression models corrected for genetic ancestry and other potential confounding factors, residual age-at-onset of HD was not significantly associated with the longer, the shorter, or the sum of the two repeat alleles of any of the tested genes (Table 1). In contrast, HD age-at-onset was significantly associated with the size of expanded *HTT* CAG repeat (*P*-value, 2.1E-111), consistent with our previous report.^12^ As expected, residual age-at-onset, representing onset age corrected for the length of expanded *HTT* CAG repeat, was not significantly associated with *HTT* CAG repeat size (expanded repeat *P*-value, 0.6948; normal repeat *P*-value, 0.7171).

**Table 1:** Statistical analysis to test association between residual age-at-onset of HD and CAG repeat size of other polyglutamine disease-associated genes

Gene	Disease	Subjects (*n*)	Range of repeat length	*P*-value		
				Longer repeat	Shorter repeat	Sum of repeats
*ATN1*	DRPLA	551	4–19	0.4691	0.1653	0.0635
*ATXN1*	SCA1	502	21–37	0.3091	0.3281	0.8652
*ATXN2*	SCA2	604	14–33	0.7241	0.9873	0.8670
*ATXN3*	SCA3	503	14–41	0.5038	0.9361	0.5517
*CACNA1A*	SCA6	497	4–18	0.5760	0.1205	0.5201
*TBP*	SCA17	483	29–42	0.837	0.452	0.3904

To test whether residual age-at-onset of HD is significantly associated with CAG repeat lengths of other polyglutamine disease-associated genes, we performed linear regression analyses. For each of the test genes, either the longer, shorter or the sum of both repeat alleles was used as a continuous predictor variable to explain residual age-at-onset of HD. Sample size and *P*-value are shown. *R*-squared values were smaller than 1% for all tested alleles.

### Association analysis of *ATXN3* CAG repeat

**Figure 1:**
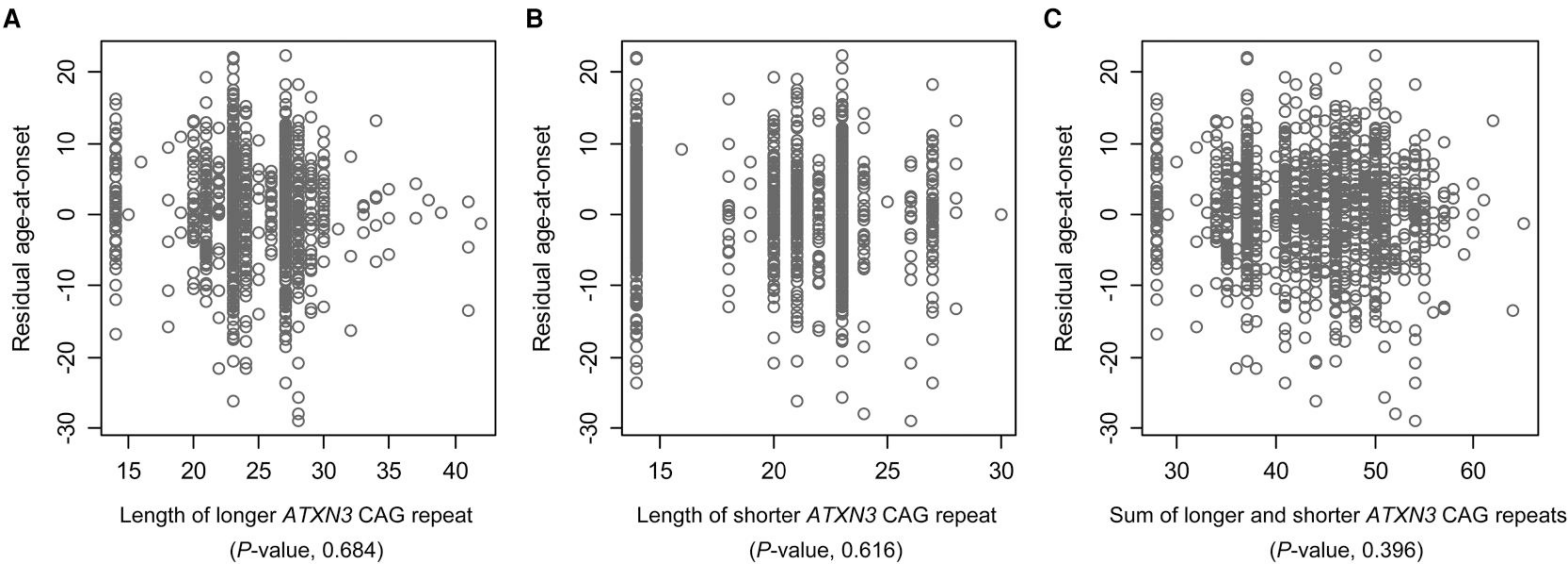
**The lack of modification of HD onset by *ATXN3* CAG repeats.** To validate the COHORT data analysis of *ATXN3* results, we also analyzed the *ATXN3* repeat in the REGISTRY samples. Subsequently, we performed linear regression analysis using the combined data to determine whether the longer **A**, shorter **B,** or sum of the two repeat alleles **C,** could explain residual age-at-onset of HD (*n* = 1388). Twenty-six repeat alleles were observed; the most and second most frequent repeat alleles were 23 and 14 CAGs, accounting for 53% of all repeat alleles. The *Y*-axis shows residual age-at-onset of HD subjects, representing age-at-onset corrected for individual *HTT* CAG repeat size (i.e. years). The *X*-axis represents the length of CAG repeat of *ATXN3* (i.e. [CAG]n).

It has been proposed that the length of CAG repeat in *ATXN3* is associated with age-at-onset of HD.^45^ In contrast, our initial analysis of the COHORT participants (*n* = 503) did not show statistically significant associations between HD residual age-at-onset and *ATXN3* CAG repeat lengths. To confirm this lack of association in a larger sample set, we also analyzed REGISTRY participants who were also part of our recent GWA study. A total of 1388 (706 males and 682 females) unique HD individuals with European ancestry (503 COHORT and 885 REGISTRY) were analyzed for *ATXN3* CAG repeats. Consistent with our initial observations, linear regression analyses to test the longer, the shorter, or the sum of the two repeats of *ATXN3* showed no statistically significant associations with residual age-at-onset of motor signs (Fig. 1). We further performed a dichotomous analysis to test whether age-at-onset or residual age-at-onset was significantly different between HD individuals carrying *ATXN3* repeats above and below the median length. As shown in Supplementary Fig. 2, these two groups of HD study participants were not significantly different for age-at-onset (Supplementary Fig. 2A) or residual age-at-onset (Supplementary Fig. 2B), arguing against modification of HD by the *ATXN3* CAG repeats.

### No SNP association signals at other polyglutamine disease-causing genes in HD onset modifier GWAS data

Previously, we discovered genetic modifiers of HD through genome-wide association analysis of SNPs,^15^ and subsequently generated a website to make these GWAS results publicly accessible.^49^ We used this resource to check HD modification signals at the polyglutamine disease-causing gene assessed above, plus spinocerebellar ataxia type 7 (SCA7, MIM #607640) and spinal and bulbar muscular atrophy (*AR,* MIM #313200). We also included *PPP2R2B* and *DMPK*, expansions of CAG•CTG repeats within which underlie spinocerebellar ataxia type 12 (SCA12, MIM #604326) and myotonic dystrophy type 1 (DM1, MIM #160900), respectively, but are not translated into polyglutamine. For each locus, we evaluated the RefSeq select (‘ncbiRefSeqSelect’ in the UCSC genome browser) as the representative transcript and identified the SNP with the smallest association *P*-value in the transcript region. The top SNPs at other CAG repeat expansion disease-causing genes were relatively infrequent except *AR.* We observed nominally significant *P*-values for the association of some loci with HD residual age-at-onset (Supplementary Table 1), but when these were corrected for the gene size and number of SNPs in the region, none remained statistically significant. Together, our HD modifier SNP GWAS data did not support modification of HD by variants in these other CAG repeat expansion disease-causing genes.

### Genome-wide STR association analysis

**Figure 2:**
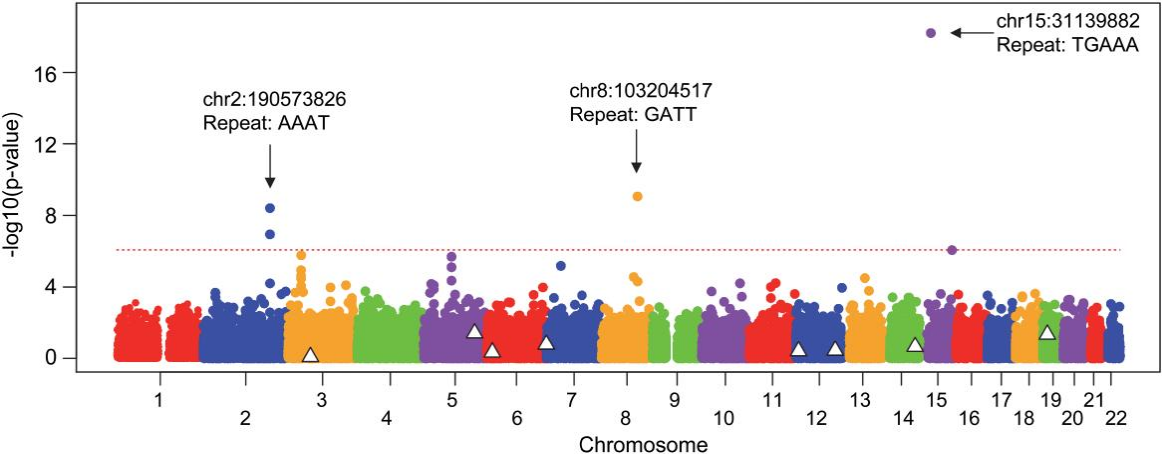
**Genome-wide STR association analysis of residual age-at-onset of HD.** STRs were imputed based on the typed SNP data and subsequently used as the predictor variable with other covariates to explain residual age-at-onset of HD. For this analysis, we used the sum of two alleles for a given STR (i.e. additive model). *Y*-axis represents significance levels of association, expressed as –log10(*P*-value). A dotted horizontal line represents Bonferroni significance (*P*-value, 8.48E-7 based on 58 894 tests). Triangles mark the polyglutamine disease-causing repeats.

Though our GWAS, representing the largest dataset of HD individuals with genome-wide genotype and phenotype,^15^ successfully identified associated SNPs, those analyses could have limited power to assess effects of polymorphic STRs on modification of HD. Recently, methods for imputation of STR lengths from genome-wide SNP data have been developed and further optimized.^51^ Therefore, in order to test the association between HD onset and repeat size of polyglutamine disease-causing genes and other STRs, we imputed ∼60 000 STRs for the HD subjects who participated in our HD modifier GWAS (*n* = 9058). We then performed association analysis using the same residual age-at-onset phenotype as in our GWAS^15^ and STR genotypes as the independent variable with a set of covariates. Specifically, we used the sum of the two repeat lengths, which is similar to the additive model in standard SNP analysis. Notably, the genomic regions containing other polyglutamine disease-causing genes showed no significant STR association signals (Fig. 2, white triangles). The imputed CAG repeat sizes of other polyglutamine disease-causing genes were also not significantly associated with HD residual age-at-onset (Table 2) based on the Bonferroni multiple correction method (*P*-value, 8.5E-7). We further evaluated the levels of association between age-at-onset of HD and imputed CAG repeat of *ATXN3*. To confirm the quality of STR imputation, we compared the genotyped and the imputed *ATXN3* CAG repeat in 1388 HD individuals where both estimates were available. The longer, the shorter, and the sum of the two alleles of the *ATXN3* repeat showed 74.6, 87.2, and 69.2% concordance between experimentally determined and imputed repeat lengths (Supplementary Fig. 3). Moreover, more than 90% of the observed differences were fewer than five repeats (Supplementary Fig. 3D), suggesting relatively high levels of accuracy in STR imputation. Like the analysis using typed data, association analyses to test the longer (Supplementary Fig. 4A), the shorter (Supplementary Fig. 4B), or the sum of the two STR alleles (data, not shown) in the imputed data did not reveal statistically significant association with HD age-at-onset. Furthermore, HD participants who carry longer *ATXN*3 repeats smaller than the median of the longer repeat (i.e. 23 CAG) showed the same age-at-onset and residual age-at-onset compared to those with longer *ATXN3* repeats larger than median (Supplementary Fig. 4C and 4D). Together, our genetic analyses using both genotyped and imputed STR data strongly indicated that *ATXN3* CAG repeat length does not modify HD age-at-onset, in contrast to the previous report.

**Table 2:** Significance of CAG repeat in imputed STR association analysis

Polyglutamine disease	Chromosomal location (GRCh37/hg19)	Gene	*P*-value
DRPLA (Dentatorubropallidoluysian atrophy)	Chr12:7045880	*ATN1*	0.5785
SCA1 (Spinocerebellar ataxia type 1)	Chr6:16327865	*ATXN1*	0.4584
SCA2 (Spinocerebellar ataxia type 2)	Chr12:112036754	*ATXN2*	0.3602
SCA3 (Spinocerebellar ataxia type 3)	Chr14:92537353	*ATXN3*	0.2198
SCA6 (Spinocerebellar ataxia type 6)	Chr19:13318673	*CACNA1A*	0.0442
SCA7 (Spinocerebellar ataxia type 7)	Chr3:63898361	*ATXN7*	0.8243
SCA12 (Spinocerebellar ataxia type 12)	Chr5:146258291	*PPP2R2B*	0.0377
SCA17 (Spinocerebellar ataxia type 17)	Chr6:170870996	*TBP*	0.1638

Significance of CAG repeat of other polyglutamine disease genes extracted from the genome-wide STR association analysis using an additive model. Since STRs were imputed only for autosomes, the spinal and bulbar muscular atrophy CAG repeat on the X chromosome was not assessed.

In contrast, as shown in Fig. 2, we identified three Bonferroni significant modification signals (*P*-value <8.5E-7) tagged by STRs on chromosome 2, chromosome 8 and chromosome 15, that potentially captured effects of modification by *PMS1*, *RRM2B* and *FAN1*,^15^ which were also implicated by SNP association. Similarly, the previously implicated *MLH1* region of chromosome 3 and the *MSH3* region at chromosome 5 showed STR association signals at suggestive significance (*P*-value, E-5). Interestingly, a near significant new signal (uncorrected *P*-value, 8.7E-7) was evident on distal chromosome 15q with a guanine-adenine repeat (chr15:91711532-91711561) in synaptic vesicle glycoprotein 2B (*SV2B*) gene, which encodes SV2B. Still, confirmation analysis by direct repeat genotyping will be required to establish their roles in modifying HD, considering the inherent uncertainty in imputing tandem repeats from SNP data.

## Discussion

The existence of multiple neurodegenerative disorders associated with lengthened polyglutamine segments in different proteins has implicated common unifying mechanisms of pathogenesis^3,4,53^ and also raised the related possibility that CAG repeat disease genes show functional interaction with one another in modulating disease manifestations. For example, in early studies, SCA3 fasciculations were reported to be associated with normal *ATXN2* CAG repeat length,^54^ while SCA2 onset was reported to be influenced by the normal *CACNA1A* CAG repeat which, when expanded, causes SCA6.^55^ Subsequently, age-at-onset in several SCAs was reported to be influenced by CAG repeat length variation in various polyglutamine disease-causing genes.^46,56,57^ There have also been interactions suggested between coding and non-coding CAG repeats, as the *ATXN1* 31 CAG repeat allele was reported to be enriched in myotonic dystrophy, which is caused by an expanded CTG repeat in the *DM1* 3′- untranslated region of the *DMPK* gene,^58^ although these findings were challenged later.^59^ Specifically in HD, age-at-onset appeared to be modified by the normal CAG repeat in *ATXN3*, which is responsible for causing SCA3 when expanded.^45^ Given the variability observed across these studies and the possibility that such genetic interactions could provide important insights into both underlying disease mechanisms and potential therapeutic directions, we reasoned that the possibility of modification of HD by other CAG repeat disease-causing genes was an important subject for rigorous investigation.

Using both a candidate approach based on CAG length and an unbiased SNP-based GWAS, we did not detect any significant influence of polyglutamine disease-causing genes and other CAG repeat expansion disease-causing genes on the age-at-onset of HD. The lack of replication of candidate modifiers of HD has been reported before. For example, candidate studies suggested modification of HD by *ADORA2A*,^60,61^  *ATG7*,^62^  *BDNF*,^63^  *GRIK2*,^64^  *GRIN2A*,^64-66^  *GRIN2B*,^64,65^  *HAP1*,^67^  *HIP1*,^64^  *LINC01559*,^64^  *NPY2R*,^68^  *PPARGC1A*^69-72^ and *UCHL1*.^73^ However, none of these genes generated significant onset modification signals in our large scale unbiased genetic analysis.^14,15,49,74^ Interestingly, one candidate modifier that showed a trend of association^66,75^ and was replicated by GWAS is *TCERG1,* which harbours a complex coding hexamer repeat that appears to be the source of the influence on HD age-at-onset.^76^ Unfortunately, the hexamer repeat with potential association was not imputed in our data because this repeat was not present in the imputation reference panel that we used. Nevertheless, the lack of replication for most candidates could be due to spurious signals from underpowered studies, confounded by ancestry differences, lack of multiple test corrections and/or outlier effects.^74,77^ Outlier effects are particularly significant when using continuous variables, as we observed that a single data point could change insignificant signal into significant association.^12^ This may explain the lack of replication of modifying effects of the *ATXN3* repeat on HD age-at-onset. The overall high rate of failure to replicate reinforces the importance of rigorous data quality control and stringent statistical analysis for the association analysis of human data.

Although our data did not validate the modification of HD by other polyglutamine disease-causing genes, imputed STR data did show Bonferroni significant signals on chromosomes 2, 8 and 15. These significant STRs appear to tag previously identified modifier haplotypes of *PMS1*, *RRM2B* and *FAN1,* which were detected in our SNP-based GWAS. Considering that this original association analysis tested more than 10 million SNPs, the detection of these three significant association signals from testing ∼60 000 genetic polymorphisms supports the levels of power and efficiency of the STR approach and argues for its use in modifier studies of other disorders. More than sixty diseases are known to be caused by expansions of tandem repeats, and additional disease-causing repeats are being discovered with the advance of genomic technologies.^78,79^ In addition to tandem repeats that cause Mendelian disorders, repeat polymorphism may contribute to the missing heritability of common polygenic disorders.^80-82^ Importantly, changes in tandem repeats represent one of the major sources of *de novo* mutation with clinical significances.^83,84^ For example, somatically expanded tandem repeats influence disease age of onset and tissue specificity of pathogenic features^78,79^ Furthermore, significant genome-wide excess of tandem repeat mutations has been reported in the autism spectrum disorder,^85-87^ implying that tandem repeats may have profound effects on human health beyond the well-characterized repeat expansion disorders. Many GWAS signals are due to effects on gene expression levels, and recent findings of a role for length and motif composition of the tandem repeats including variable number tandem repeats (VNTRs) in regulating gene expression^88-92^ suggest that some HD onset modification signals might be caused by altered expression of modifier genes due to polymorphic tandem repeat lengths. Therefore, investigating a potential role for tandem repeats in regulating the expression levels of the *PMS1, RRM2B*, and *FAN1* modifier genes implicated at the chromosome 2, 8 and 15 loci may reveal an important underlying source of HD modification. Finally, the STR association signal on distal chromosome 15q suggests the possibility of an HD modifier effect due to *SV2B*. The *SV2B* protein localizes to synaptic vesicles, where it is believed to function in the regulation of vesicle trafficking and exocytosis. While a role at synapses makes it an attractive candidate for involvement in the HD damage mechanism(s) precipitated by the expanded CAG repeat, this locus will require replication or other confirmation to ensure that it harbours a *bona fide* genetic modifier of HD.

In summary, we expanded our approaches for identifying genetic modifiers of HD using typed and imputed repeats. We focused on STRs in this study primarily due to their clinical significance and available resources for genome-wide imputation.^51,78,79,84^ However, other genetic variations (i.e. VNTR and structural variations) also generate biological consequences in humans.^93,94^ Therefore, investigation of other types of DNA polymorphisms may yield a more complete map of genetic modifiers of HD. Nevertheless, although our data clearly show the lack of modification of HD onset by CAG repeat size polymorphisms in other polyglutamine disease genes, they do point, along with the complex coding hexamer repeat in *TCERG1*^76^ and a complex nonamer coding repeat in *MSH3*,^21^ to the potential of finer delineation of other tandem repeats across the genome as a potential source of modifiers that could further refine the HD landscape and inform the development of treatments for HD.

## Supplementary Material

fcae016_Supplementary_Data

## Data Availability

STR GWAS summary data that support the findings of this study are available from the corresponding author, upon reasonable request.
