# Supplementary material for: Modification of Huntington’s disease by short tandem repeats
Source: Brain Commun. 2024 Jan 23;6(2):fcae016. doi: 10.1093/braincomms/fcae016 (PMC10917446; doi:10.1093/braincomms/fcae016)
Supplement: fcae016_Supplementary_Data [file fcae016_supplementary_data.pdf]

Supplementary Table 1. SNP association with residual age-at-onset in HD.

For each of other CAG repeat expansion disease genes, we evaluated the levels of association based on our recent SNP association for HD modification. A RefSeq select transcript as a representative transcript was used for a given region to identify the SNP with the highest significance in the GeM Euro 9K website. To correct the top SNP *P*-value for the gene size and number of SNPs we applied modified Sidak method to obtain corrected *P*-value.

Gene	Disease	Top SNP	MAF (%)	*P*-value	Corrected *P*-value
ATN1	DRPLA	rs181318837	1.539	0.036365	0.296653
AR	SBMA	rs5918762	15.58	0.372432	1
ATXN1	SCA1	rs80281835	1.677	0.003874	0.937191
ATXN2	SCA2	rs77838113	2.609	0.016352	0.599493
ATXN3	SCA3	rs55961283	2.499	0.103693	0.999995
ATXN7	SCA7	rs77203794	1.473	0.025268	0.952429
CACNA1A	SCA6	rs145803932	1.749	0.017769	0.99987
PPP2R2B	SCA12	rs4705448	1.114	0.000421	0.207854
TBP	SCA17	rs73256671	6.509	0.039786	0.598874
DMPK	DM1	rs183029748	1.269	0.072635	0.492709

Supplementary Figure 1. Distributions of CAG repeats of polyglutamine disease-causing genes in HD subjects.

Distributions of CAG repeats at both alleles for each polyglutamine disease-causing gene are plotted. The sizes of CAG repeats of other polyglutamine disease-causing genes were determined in 483 to 604 HD individuals (Table 1). For each of other polyglutamine disease gene, mean and standard deviation was used to generate a theoretical normal distribution to plot the patterns of CAG repeat length distribution. The grey background histogram represents *HTT* CAG repeat lengths.

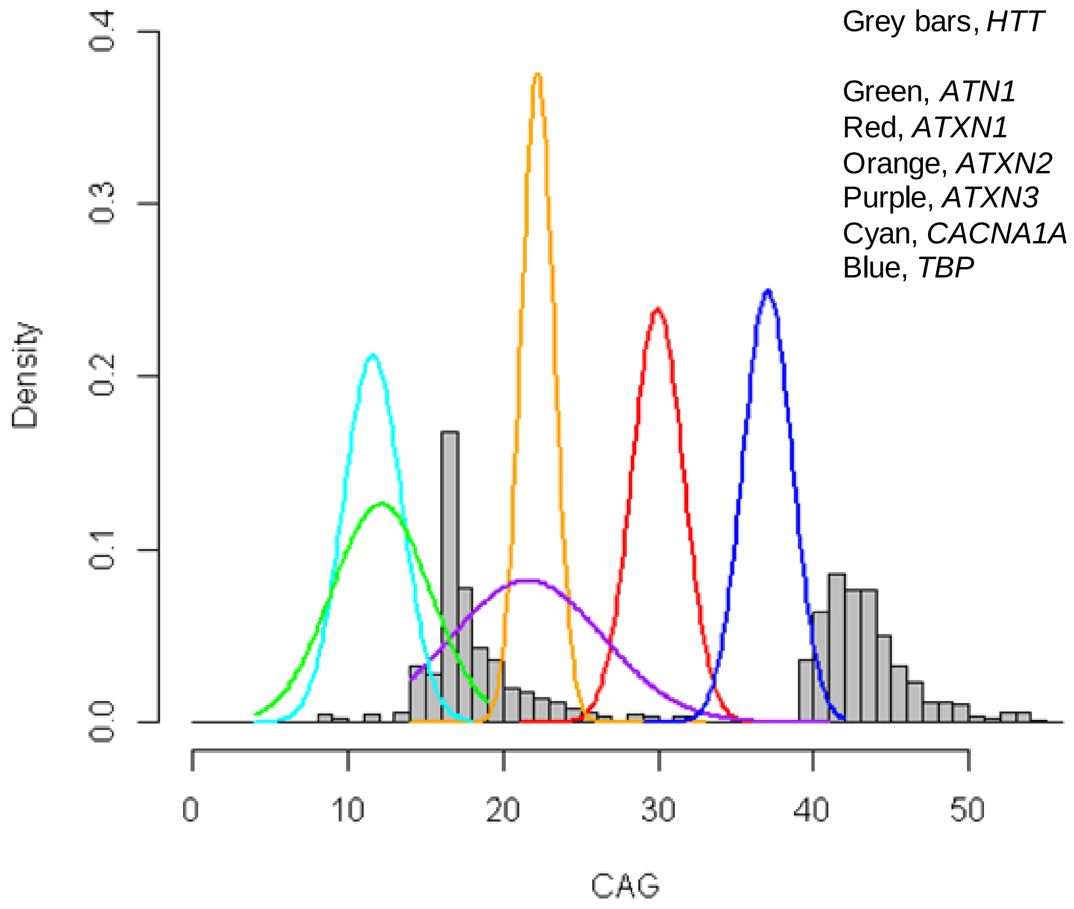

Supplementary Figure 2. Same age-at-onset and residual age-at-onset between HD subjects carrying below and above CAG repeat of *ATXN3*.

HD subjects were grouped based on the median of longer allele of the *ATXN3* CAG repeat (i.e., 23), generating a below the median group (*ATXN3* CAG < 23) and an above the median group (ATXN3 CAG > 23). Subsequently, age-at-onset (A) and residual age-at-onset (B) were compared between two groups using Mann-Whitney *U*-test. *P*-values are shown at the bottom of each plot.

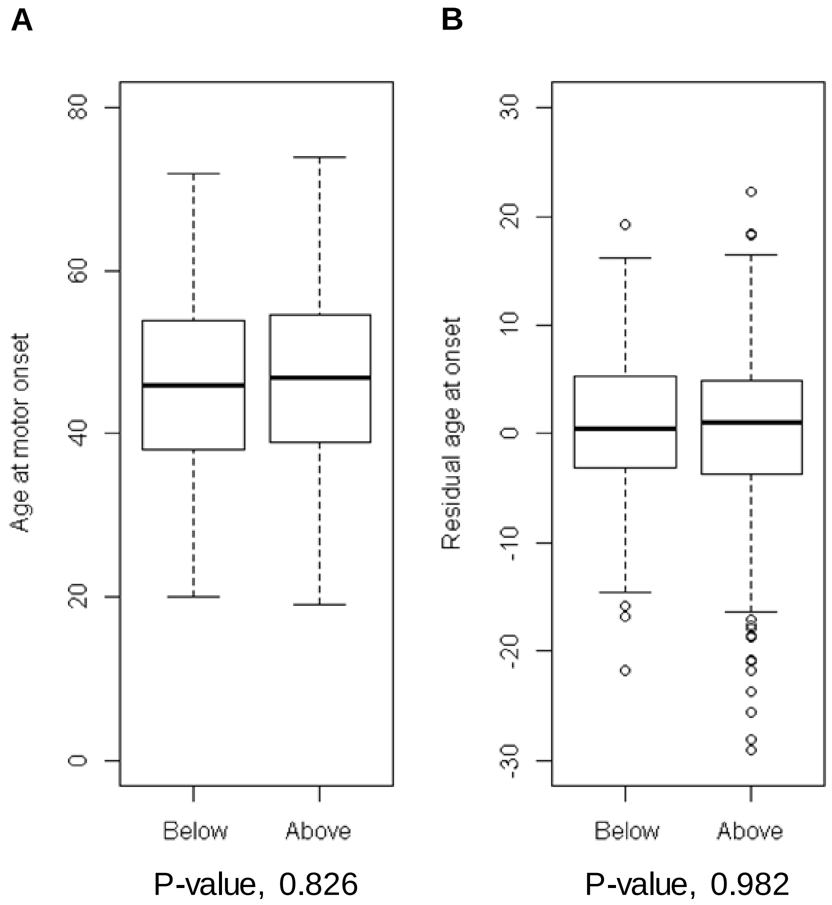

Supplementary Figure 3. Correlation between genotyped and imputed *ATXN3* CAG repeats.

To evaluate the accuracy of STR imputation, we compared *ATXN3* CAG repeats that were experimentally determined and imputed. A total of 1388 samples had both typed and imputed *ATXN3* CAG repeats. We compared longer repeat (A), shorter repeat (B), and the sum of two repeats (C) in imputed data (Y-axis) and typed data (X-axis). We also calculated percentage of identical or similar (difference smaller than 5) were calculated for longer, shorter and sum (D).

A B C

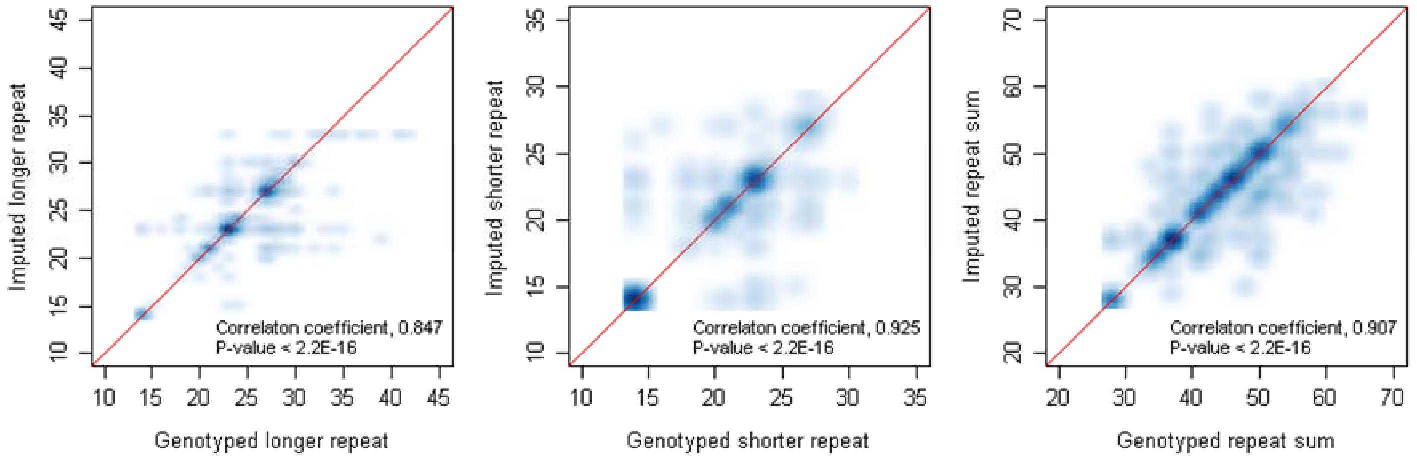

D

	Longer repeat	Shorter repeat	Sum
Identical between genotyped and imputed repeats	74.6%	87.2%	69.2%
Difference between genotyped and imputed repeats < 5	93.8%	95.4%	93.8%

Supplementary Figure 4. The lack of association between HD residual age-at-onset and imputed *ATXN3* CAG repeats

Genome-wide STR association analysis used sum of the two alleles for each STR, arguing against modification of HD by *ATXN3* repeat. We further tested whether imputed longer allele (A) or imputed shorter allele (B) is associated with residual age-at-onset. Also, we compared age-at-onset (C) and residual age-at-onset (D) between HD subjects with below and above median repeat length. Respective *P*-values are shown below of each plot.

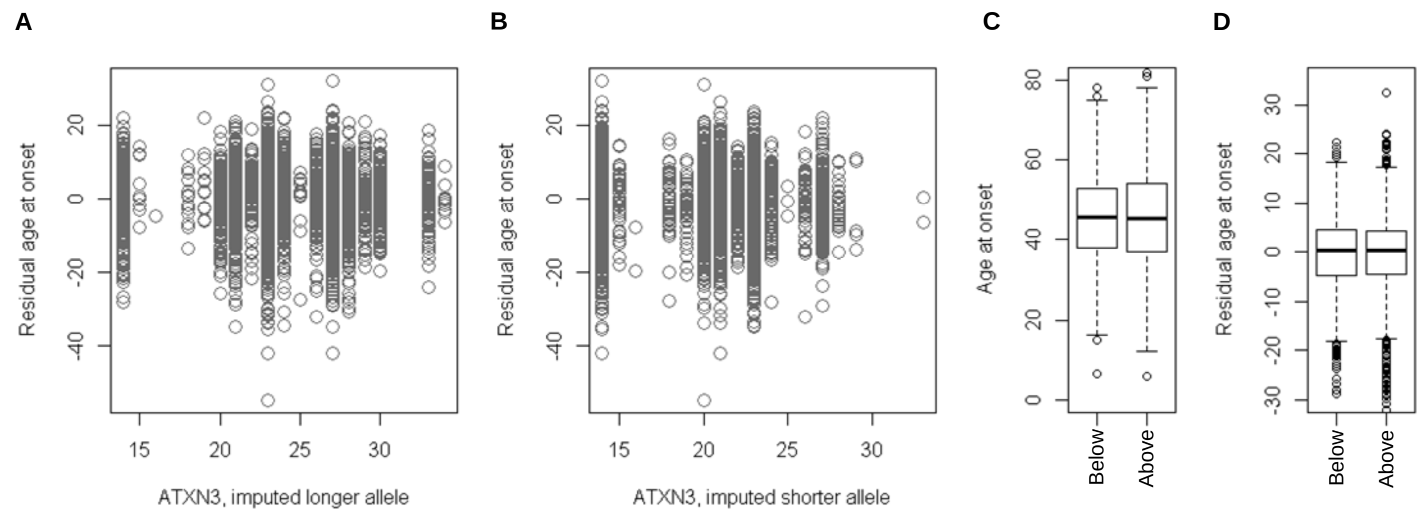

P-value, 0.268 P-value, 0.798 P-value, 0.946 P-value, 0.666
